# Exogenous Melatonin Modulates the Physiological and Biochemical Mechanisms of Drought Tolerance in Tartary Buckwheat (*Fagopyrum tataricum* (L.) Gaertn)

**DOI:** 10.3390/molecules25122828

**Published:** 2020-06-18

**Authors:** Md. Shakhawat Hossain, Jing Li, Ashim Sikdar, Mirza Hasanuzzaman, Ferdinand Uzizerimana, Izhar Muhammad, Yuhao Yuan, Chengjin Zhang, Chenyang Wang, Baili Feng

**Affiliations:** 1 College of Agronomy, Northwest A&F University, Yangling 712100, Shaanxi, China; shaiful27917@gmail.com (M.S.H.); lijing1993@nwafu.edu.cn (J.L.); uziferd@gmail.com (F.U.); izeyaar@gmail.com (I.M.); yuanyuhao@nwafu.edu.cn (Y.Y.); chengjinzxx@163.com (C.Z.); wcy410223@163.com (C.W.); 2 College of Natural Resources and Environment, Northwest A&F University, Yangling 712100, Shaanxi, China; ashim.aes@sau.ac.bd; 3 Department of Agroforestry and Environmental Science, Sylhet Agricultural University, Sylhet 3100, Bangladesh; 4 Department of Agronomy, Faculty of Agriculture, Sher-e-Bangla Agricultural University, Dhaka 1207, Bangladesh; mhzsauag@yahoo.com

**Keywords:** tartary buckwheat, drought stress, exogenous melatonin, antioxidant activity, secondary metabolites, reactive oxygen species, drought tolerance

## Abstract

Tartary buckwheat is one of the nutritious minor cereals and is grown in high-cold mountainous areas of arid and semi-arid zones where drought is a common phenomenon, potentially reducing the growth and yield. Melatonin, which is an amphiphilic low molecular weight compound, has been proven to exert significant effects in plants, under abiotic stresses, but its role in the Tartary buckwheat under drought stress remains unexplored. We evaluated the influence of melatonin supplementation on plant morphology and different physiological activities, to enhance tolerance to posed drought stress by scavenging reactive oxygen species (ROS) and alleviating lipid peroxidation. Drought stress decreased the plant growth and biomass production compared to the control. Drought also decreased Chl a, b, and the Fv/Fm ratio by 54%, 70%, and 8%, respectively, which was associated with the disorganized stomatal properties. Under drought stress, H_2_O_2_, O_2_^•−^, and malondialdehyde (MDA) contents increased by 2.30, 2.43, and 2.22-folds, respectively, which caused oxidative stress. In contrast, proline and soluble sugar content were increased by 84% and 39%, respectively. However, exogenous melatonin (100 µM) could improve plant growth by preventing ROS-induced oxidative damage by increasing photosynthesis, enzymatic antioxidants (superoxide dismutase, peroxidase, catalase, and ascorbate peroxidase), secondary metabolites like phenylalanine ammonialyase, phenolics, and flavonoids, total antioxidant scavenging (free radical DPPH scavenging), and maintaining relative water content and osmoregulation substances under water stress. Therefore, our study suggested that exogenous melatonin could accelerate drought resistance by enhancing photosynthesis and antioxidant defense in Tartary buckwheat plants.

## 1. Introduction

Various factors are responsible for the smooth regulation of morphological, physiological, biochemical, and metabolic processes in the plant body. Water is the most vital factor among them and, thus, affects plant development and crop yield, to a great extent [1,2]. The osmotic stress caused by drought primarily reduces plant water uptake and fuels many physiological and biochemical imbalances, resulting in stomatal closure, impaired photosynthesis, reduced transpiration, decline of cellular energy, and subsequently increases oxidative stress [2,3]. Nevertheless, plants develop an antioxidant defense system as a safeguard against the detrimental impacts of reactive oxygen species (ROS) under any kind of environmental stress. The collapse of the plant’s antioxidant defense system might occur if the level and duration of stress exceed the plant’s defense ability [4]. Excessive ROS accumulation might result in oxidation of proteins, lipids, and DNA [5]. Plant antioxidant defense mechanisms include enzymatic antioxidants like peroxidase (POD), superoxide dismutase (SOD), catalase (CAT), ascorbate peroxidase (APX), and enzymes of the ascorbate-glutathione (AsA-GSH) cycle, as well as non-enzymatic antioxidants like glutathione (GSH), ascorbate (AsA), carotenoids, tocopherols, flavonoids, and phenolics [6,7,8]. In plants, ROS mainly include O_2_^•−^, OH^•^, and H_2_O_2_. The antioxidant enzyme of SOD can deoxidize O_2_^•−^ to H_2_O_2_, which is further detoxified to H_2_O by other enzymes like CAT, APX, and POD [6,9,10]. Apart from the antioxidant defense, plants also accumulate beneficial metabolites like proline (Pro) and soluble sugar in cells by altering the primary and secondary metabolism and thus promoting the retention of water to adapt to the dry environment [11]. Secondary metabolites like phenolic compounds also play a vital role in the defense mechanism against ROS-induced damage, and their production is affected by environmental stresses like drought [12]. Phenylalanine ammonialyase (PAL) and polyphenol peroxidase (PPO) production enhance and strengthen the plant defense system, regulating plant growth [13].

Use of bioregulators or bio-stimulants to facilitate plant growth and to boost drought tolerance has emerged as an effective and eco-friendly approach [14,15]. Melatonin (N-acetyl-5-methoxytryptamine) is a kind of biological regulator, which can positively affect plant growth and development by modulating different physiological and biochemical mechanisms [16]. It can regulate photosynthesis as well as maintain redox homeostasis by improving the activities of antioxidant enzymes under abiotic stresses [16,17]. Campos et al. [18] observed that melatonin pretreatment could improve stress tolerance and inhibit ROS-induced oxidative damage by regulating the expression of stress response genes. Recently, Sharma and Zheng [19] indicated that melatonin protects plants from the adverse effects of drought-induced oxidative stress by enhancing the ROS scavenging efficiency. A few studies have reported that melatonin might facilitate or inhibit plant flowering as well as the maturation of plants [20,21]. It is hypothesized that those functions were in fact acquired during evolution [22], but they were not the original purpose of melatonin in plants. In addition, melatonin being a nontoxic biodegradable molecule, is highly recommended for promoting environment-friendly crop production [23]. Although the role of melatonin in stress tolerance of crop plants has been reported in several studies, there is a scarcity of reports regarding its role in modulating phenolic compounds, osmolytes, and phenolic enzymes like PAL and PPO for melatonin application under drought stress.

Tartary buckwheat (*Fagopyrum tataricum* (L.) Gaertn) is one of the minor cereals in Asia, Europe, North America, and South Africa, belonging to the family Polygonaceae and genus *Fagopyrum*. In recent times, Tartary buckwheat is mainly consumed in the form of noodles in Japan, Italy, and China. However, the seeds are also widely used as buckwheat flour, bread, vinegar, tea, and sprouts for centuries [24]. As the Tartary buckwheat is rich in vitamins, minerals, dietary fiber, proteins, amino acids, various bioactive phytochemicals, and trace elements, the interest in its use for health benefits is increasing, and the market demand is also gradually rising [25]. It is mainly grown in high-cold mountainous areas of arid and semi-arid zones where precipitation is remarkably infrequent [26], but now it is also cultivated in the plain lands of different countries, because of the growing consumption rate. Therefore, the inadequacy of water is one of the important barriers to achieve the desired yield in Tartary buckwheat production all over the world. Considering the lack of study in elucidating the function of melatonin in response to environmental stresses in minor crops, especially Tartary buckwheat, we explored the effect of melatonin in conferring drought tolerance in Tartary buckwheat plants.

## 2. Results

### 2.1. Effect of Exogenous Melatonin on Plant Growth Parameters and Relative Leaf Water Content

**Figure 1:**
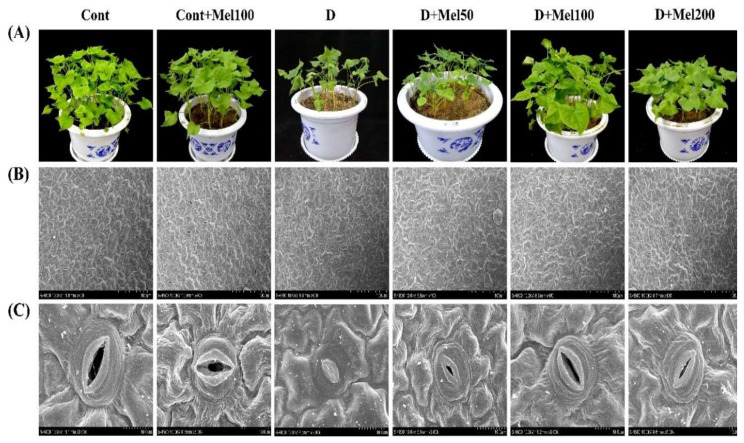
Effects of exogenous melatonin on phenotypic expression (**A**), stomatal properties of Tartary buckwheat leaves under drought stress (**B** and **C**) and magnification 400×, scale bar = 100 µm (**B**), and magnification 3000×, scale bar = 10 µm (**C**). Here, Cont: Control, 80% field capacity (FC); D: Drought, 20% FC; Mel50: 50 µM melatonin; Mel100: 100 µM melatonin, and Mel200: 200 µM melatonin.

Under drought stress, all growth parameters such as shoot height, root length, number of leaves, shoot fresh weight (SFW), root fresh weight (RFW), shoot dry weight (SDW), root dry weight (RDW), stem girth, and relative water content (RWC), efficiently (*p* ≤ 0.05) decreased by 2.03, 1.88, 2.26, 4.07, 4.99, 4.0, 4.48, 4.04, 1.84, and 1.30-folds, respectively, as compared to the control (Table 1). Application of exogenous melatonin improved plant growth under drought stress, with an increase in melatonin concentration. Especially in treatment with 100 µM melatonin application, shoot height, root length, leaf number, SFW, RFW, SDW, RDW, stem girth, and RWC were significantly (*p* ≤ 0.05) increased by 1.71, 1.61, 1.38, 3.04, 4.29, 3.06, 3.6, 3.1, 1.53, and 1.22-folds, respectively, as compared to drought treatment. In addition, plant under drought stress caused leaf deformation, and wilting of seedling were detected (Figure 1). In addition, the foliar application of melatonin in different concentrations (50–200 µM) conspicuously reduced the damaging effect of drought in the Tartary buckwheat plant, and the highest recovery was observed with 100 µM melatonin foliar application (Figure 1). On the other hand, in Cont + Mel100 treatments, plants had no noteworthy effect on growth parameters and RWC (Table 1, Figure 1A). Pearson’s correlation analysis indicated that all growth parameters had significant (*p* ≤ 0.01) positive correlation with chlorophyll (Chl) pigment, Fv/Fm values, RWC, and stomatal properties, except stomatal density, whereas a negative correlation was observed with H_2_O_2_, O_2_^•−^, MDA, PPO, and Fo, at 1% significant level.

### 2.2. The Influence of Melatonin Supplementation on Photosynthesis

**Figure 2:**
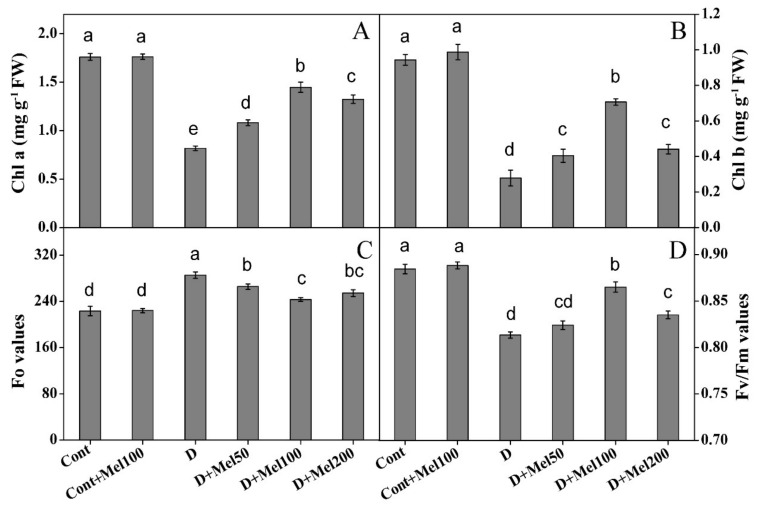
The effect of melatonin on (**A**) chlorophyll a, (**B**) chlorophyll b, (**C**) FO values, (**D**) Fv/Fm values in Tartary buckwheat under drought stress. Here, Cont: Control, 80% field capacity (FC); D: Drought, 20% FC; Mel50: 50 µM melatonin; Mel100: 100 µM melatonin, Mel200: 200 µM melatonin, Chl a: chlorophyll a, and Chl b: chlorophyll b. Values of all data are represented as mean ± standard error with three replications. Means values indicated with different letter refer to statistically significant differences at *p* ≤ 0.05, according to Duncan’s multiple range test.

The plants under drought stress showed a reduction in Chl a, b, and Fv/Fm ratio by 54%, 70%, and 8%, respectively, whereas in the control, the Fo value was enhanced by 22% (Figure 2). In contrary, plants in all melatonin treatments exhibited noticeable (*p* ≤ 0.05) diminution of the antagonistic effects of drought stress. Plants treated with 100 µM melatonin showed the highest contents of the Chl a (77%), Chl b (153%), and Fv/Fm (6%) ratio, compared to plants under drought treatment, whereas the Fo value decreased by 15% (Figure 2). In melatonin treatments in control plants, no significant effect was observed in Chl content and fluorescence (Figure 2). In addition, Chl a & b, and the Fv/Fm ratio showed a significant (*p* ≤ 0.01) negative correlation with H_2_O_2_, O_2_^•-^, MDA, and PPO, whereas a significant (*p* ≤ 0.01) positive correlation was observed with the soluble protein, RWC, and stomata properties, except for the stomatal density.

### 2.3. Impact of Melatonin Supplementation on Stomatal Properties under Drought Stress

**Figure 3:**
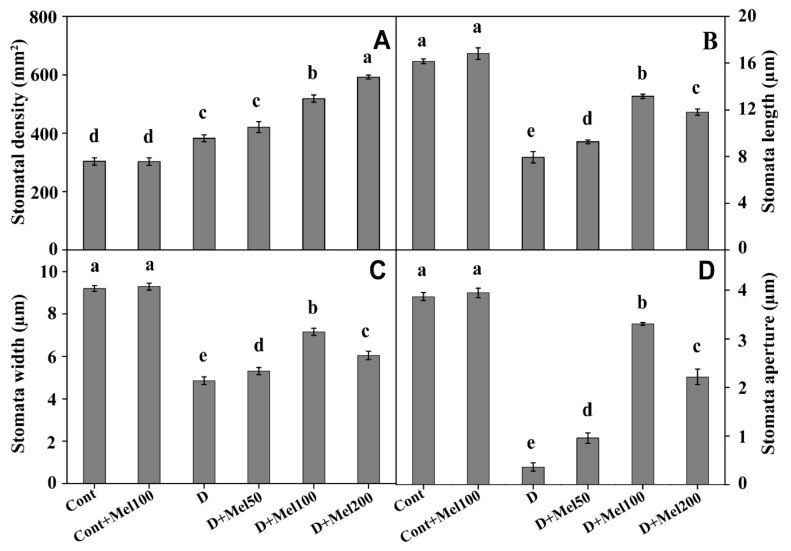
Impacts of melatonin supplementation on stomatal density (**A**), stomata length (**B**), stomata width (**C**) and stomata aperture (**D**) of Tartary buckwheat plants under drought stress. Here, Cont: Control, 80% field capacity (FC); D: Drought, 20% FC; Mel50: 50 µM melatonin; Mel100: 100 µM melatonin, and Mel200: 200 µM melatonin. Values of all data are represented as mean ± standard error with three replications. Means values indicated with different letters refer to statistically significant differences at *p* ≤ 0.05, according to Duncan’s multiple range test.

Drought stress significantly (*p* ≤0.05) decreased length, width, and aperture of stomata by 2.04, 1.9, and 10.71-folds, respectively, whereas it increased stomatal density (1.26-fold) over the control. In contrary, all treatments with melatonin supplementation increased length, width, aperture, and density of stomata under drought-stressed plants (Figure 1B,C and Figure 3). Among these treatments, 100 µM melatonin in drought stress (D + Mel100) showed the highest enhancement in length, width, and aperture of stomata by 1.66, 1.48, and 9.17-folds, respectively, as compared to drought treatment, but the maximum increase in stomata density (1.55-fold) was observed in D + Mel200 treatments (Figure 1B,C and Figure 3). Nevertheless, melatonin addition did not alter the stomatal properties of Tartary buckwheat leaves in the absence of drought stress.

### 2.4. Impact of Foliar Application of Melatonin on Osmotic Solutes Content under Drought Stressed Plants

**Figure 4:**
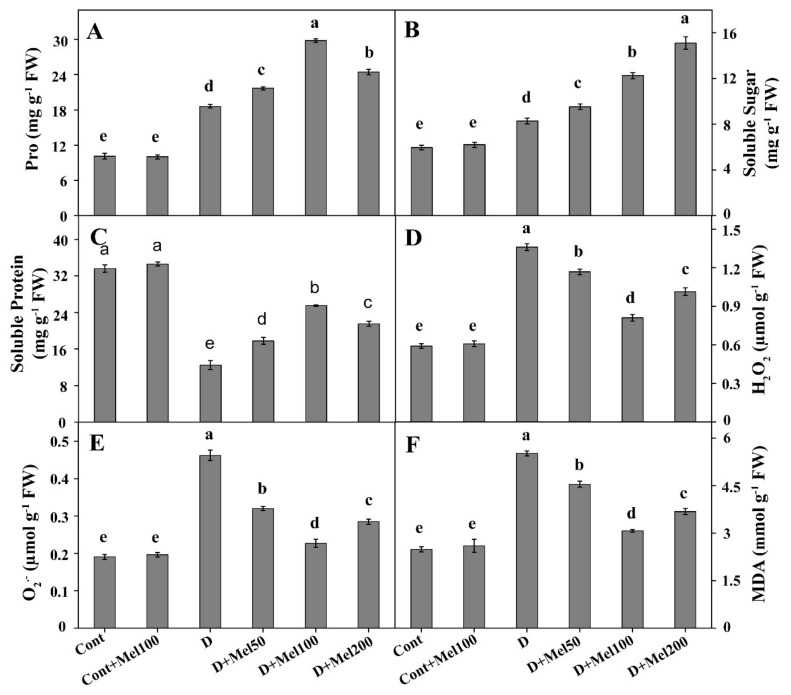
Changes in H_2_O_2_ (**A**), O_2_^•−^ (**B**), MDA (**C**), Proline (Pro) (**D**), Soluble sugar (**E**), and Soluble protein (**F**) in Tartary buckwheat leaves, after melatonin application under drought stress. Here, Cont: Control, 80% field capacity (FC); D: Drought, 20% FC; Mel50: 50 µM melatonin; Mel100: 100 µM melatonin, and Mel200: 200 µM melatonin. Values of all data are represented as mean ± standard error with three replications. Means values indicated with different letters refer to statistically significant differences at *p* ≤ 0.05, according to Duncan’s multiple range test.

All osmotic solute content (Pro, soluble sugar and soluble protein) changed under drought stress. Figure 4A,B shows that osmotic solutes like Pro and the soluble sugar content were significantly (*p* ≤ 0.05) increased under drought stress, by 84% and 39%, respectively, in comparison with control. Interestingly, Pro and soluble sugar contents increased in all melatonin treatments under drought stress over sole drought treatment (Figure 4A,B). The highest Pro content was estimated in D + Mel100 treatment, which was 60% higher than the drought-treated plants, whereas the maximum soluble sugar content was calculated in D + Mel200 treatment, which was 82% higher than that of the control. In contrast, the soluble protein content under drought stress in the buckwheat plant decreased by 63%, relative to the control (Figure 4C). In addition, the application of exogenous melatonin augmented soluble protein by 104%, as compared to the drought stress treatment (Figure 4C) and the Cont + Mel100 treatment had no significant effect on all osmotic solutes. Such results suggested that exogenous melatonin increased the content of tested osmotic solutes under drought stress in Tartary buckwheat but had no significant effect on all osmotic solutes. Such results suggested that exogenous melatonin increased the content of tested osmotic solutes under drought stress in Tartary buckwheat.

### 2.5. Effect of Exogenous Melatonin on ROS Activity and MDA Content 

Under drought stress, H_2_O_2_, O_2_^•−^, and MDA concentration significantly (*p* ≤ 0.05) increased by 2.30, 2.43, and 2.22-folds, respectively, in comparison to the control (Figure 4D–F). However, compared to the drought-stressed plants, all melatonin treatments under drought stress noticeably reduced H_2_O_2_, O_2_^•−^, and MDA concentration. Meanwhile, among the melatonin treatments, 100 µM melatonin resulted in the maximum reduction of H_2_O_2_, O_2_^•−^, and MDA concentration by 1.68, 2.04, and 1.8-folds, respectively, as compared to the drought-stressed plants (Figure 4D–F). In contrary, Cont + Mel100 treatments had no significant effect on H_2_O_2_, O_2_^•–^, and MDA. Our data indicated that melatonin supplementation impaired the accumulation of H_2_O_2_, O_2_^•–^, and MDA under drought stress in Tartary buckwheat plants.

### 2.6. Effect of Exogenous Melatonin on Enzymatic Antioxidant Activities 

**Figure 5:**
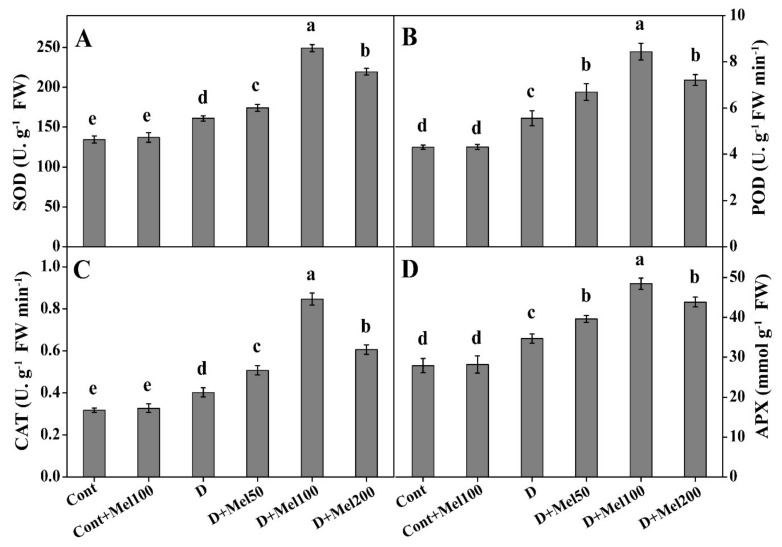
The effect of melatonin on enzymatic antioxidants like superoxide dismutase (SOD) (**A**), peroxidase (POD) (**B**), catalase (CAT) (**C**), and ascorbate peroxidase (APX) (**D**), in terms of activities in Tartary buckwheat under drought stress. Here, Cont: Control, 80% field capacity (FC); D: Drought, 20% FC; Mel50: 50 µM melatonin; Mel100: 100 µM melatonin, and Mel200: 200 µM melatonin. Values of all data are represented as mean ± standard error with three replications. Means values indicated with different letters refer to statistically significant differences at *p* ≤ 0.05, according to Duncan’s multiple range test.

To explore the impact of melatonin application on antioxidant activities in Tartary buckwheat under drought stress, we measured SOD, POD, CAT, and APX concentration. The activity of antioxidant enzymes was positively correlated with the ROS content in plant leaves. In our study, we observed that all measured antioxidant enzymatic activities significantly (*p* ≤ 0.05) increased under drought stress (Figure 5). Different concentrations of applied melatonin enhanced the activities of tested antioxidants in plants under water stress (Figure 5). The values of SOD, POD, CAT, and APX activities enhanced to the highest by 55%, 26%, 111%, and 40%, respectively, in D + Mel100 treatment, as compared to plants under drought stress (Figure 5). In addition, Pearson’s correlation analysis showed that theses antioxidant enzymes were positively correlated with H_2_O_2_, O_2_^•–^, MDA, Pro, soluble sugar, and stomatal density. Whereas, these antioxidant enzymes showed a highly positive correlation with each other at 1% significance level.

### 2.7. Impact of Melatonin Supplementation on Enzymatic and Non-Enzymatic Secondary Metabolites and Total Antioxidant Capacity

**Figure 6:**
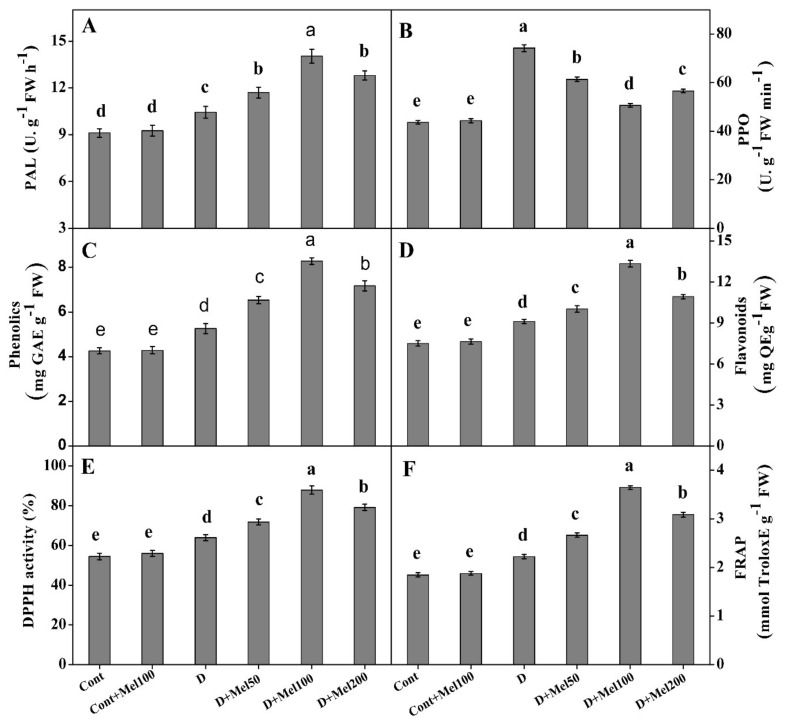
The effect of foliar applied melatonin on phenylalanine ammonialyase (PAL) (**A**), polyphenol peroxidase (PPO) (**B**), phenolics (**C**), flavonoids (**D**), DPPH (Free DPPH radical scavenger) (**E**), and ferric reducing antioxidant power (FRAP) (**F**) activity in Tartary buckwheat leaves under drought stress. Here, Cont: Control, 80% field capacity (FC); D: Drought, 20% FC; Mel50: 50 µM melatonin; Mel100: 100 µM melatonin, and Mel200: 200 µM melatonin. Values of all data are represented as mean ± standard error with three replications. Means values indicated with different letters refer to statistically significant differences at *p* ≤ 0.05, according to Duncan’s multiple range test.

As illustrated in Figure 6A and B, the activities of phenolic antioxidant enzymes such as, PAL and PPO, was effectively enhanced under drought stress in relation to the control. In all melatonin treatments, PAL activity increased, while PPO activity declined with respect to the control. Compared to drought-treated plants, the highest enhancement in PAL (34%)—and compared to the drought stress the maximum decline in PPO (32%) activity—were observed in D + Mel100 treatment plants (Figure 6A,B). The impact of melatonin on drought-stressed plants was investigated by assessing the total phenol and flavonoid contents as non-enzymatic activities in Tartary buckwheat leaves. Figure 6C,D showed that the total phenolic and flavonoid content were significantly (*p* ≤ 0.05) increased in drought-stressed plants in relation to the control. Eventually, the total phenolic and flavonoid content was found to be enhanced in plants grown in all drought treatments involving melatonin. Among the melatonin treatments, the highest total phenolic (58%) and flavonoid content (46%) were observed in D + Mel100 treatment, when compared to the control (Figure 6C,D).

**Figure 7:**
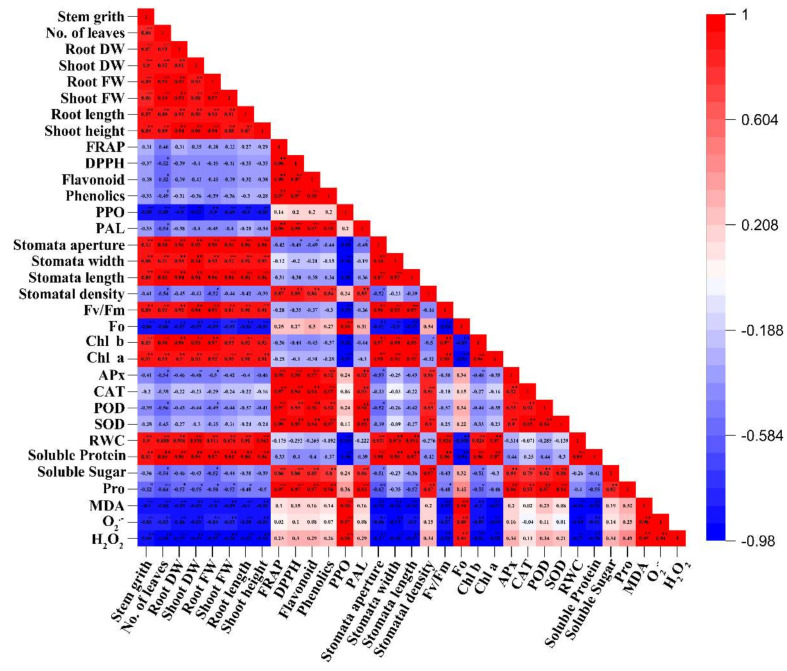
The Pearson‘s correlation analysis performed among reactive oxygen species (ROS), MDA, plant growth parameters, chlorophyll pigments, photosynthesis rate, stomata properties, and different physiological trials, after melatonin treatment under drought stress. Here, SFW—shoot fresh weight, SDW—shoot dry weight, RFW—root fresh weight, RDW—root dry weight, Chl a—chlorophyll a, Chl b—chlorophyll b, RWC—relative water content, Pro—proline, **—indicates 1% significant (*p* ≤ 0.01), and *—indicates 5% significant (*p* ≤ 0.05).

Total antioxidant capacity was investigated by estimating free radical DPPH scavenging activity and FRAP assay. The data presented in Figure 6 showed that radical DPPH scavenging and FRAP capacity significantly increased under drought stress, with respect to the control plants. Different concentrations of exogenous melatonin increased the DPPH and FRAP capacity under drought stress conditions. In DPPH assay, the antioxidant capacity was significantly high in the D + Mel100 treatment, with EC_50_ values of 3.787 µg mL^−1^, and was comparable to the EC_50_ value of the control (6.132 µg mL^−1^). Likewise, the other treated samples (Cont + Mel100, D, D + Mel50, and D + Mel200) were also compared with the control, and had EC_50_ values of 5.813, 5.162, 4.614, 4.173, and 3.787 µg mL^−1^, respectively (Supplementary Figure S1). The maximum enhancement of the DPPH (38%) and FRAP (49%) capacity was estimated in plants treated with drought alone over the control plants (Figure 6E,F), whereas the Cont + Mel100 had no significant effect on the non-enzymatic activities, phenolic enzyme activities, and total antioxidant capacity. In addition, non-enzymatic antioxidants and total antioxidant showed significant (*p* ≤ 0.01) positive correlation with SOD, POD, CAT, APX, soluble sugar, and stomatal density (Figure 7).

### 2.8. Evaluation of Melatonin Effects Under Drought Stress by Heatmap Hierarchical Clustering and Principal Component Axis Analysis

**Figure 8:**
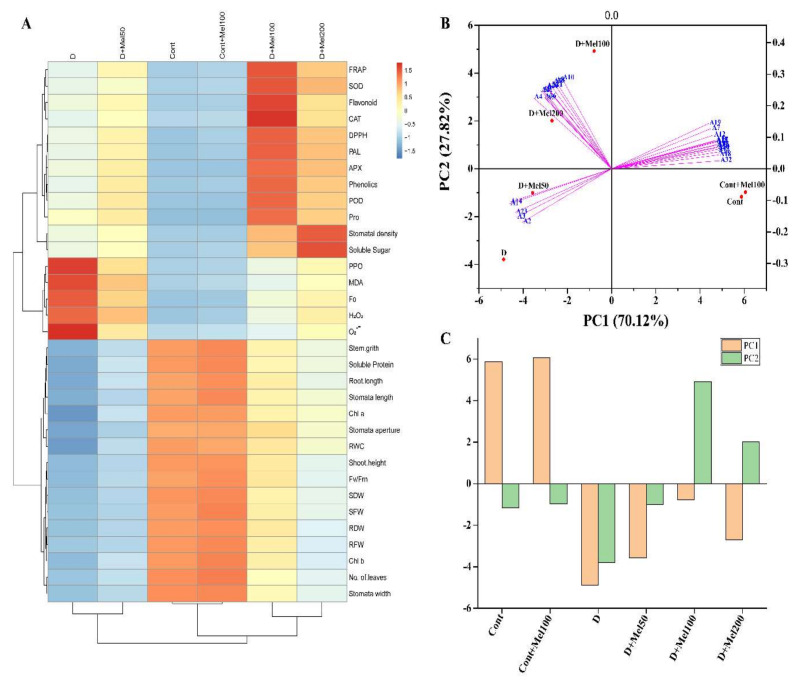
The effects of exogenous melatonin were evaluated by hierarchical clustering analysis using plant growth parameters, Chl pigments, photosynthesis rate, stomata properties, RWC, ROS, MDA, different enzymatic and non-enzymatic antioxidants, osmoregulate substances, and total antioxidant. (**A**). Principal component analysis (PCA) was performed between drought stress and melatonin treatments, with different morphological and physiological trials (**B**). Principal component analysis scoring plot among all treatment (**C**). Here, the red and blue colors indicate increase and decrease in the values of different parameters, Cont: Control, 80% field capacity (FC); D: Drought, 20% FC; Mel50: 50 µM melatonin; Mel100: 100 µM melatonin, and Mel200: 200 µM melatonin. SFW: shoot fresh weight, SDW: shoot dry weight, RFW: root fresh weight, RDW: root dry weight, Chl a: chlorophyll a, Chl b: chlorophyll b, RWC: relative water content, Pro: proline, A1- Hydrogen peroxide (H_2_O_2_), A2- Superoxide anion (O_2_^•−^), A3- MDA, A4- proline, A5- soluble sugar, A6- soluble protein, A7- SOD, A8- POD, A9- CAT, A10- APX, A11- Chl a, A12- Chl b, A13- Chl b, A14- Fo, A15- Fv/Fm, A16- phenolics, A17- flavonoids, A18- DPPH, A19- FRAP, A20- PAL, A21- PPO, A22- stomatal density, A23- stomata length, A24- stomata width, A25- stomata aperture, A26- shoot height, A27- root length, A28- shoot fresh weight, A29- root fresh weight, A30- shoot dry weight, A31- root dry weight, A32- total dry biomass, A33- stem girth, and A34- RWC.

Heatmap analysis revealed that drought stress efficiently decreased all plant growth parameters, RWC, stomata properties (except stomatal density), Chl pigments, Fv/Fm values, and soluble protein, whereas the highest value of these trials was mostly found in both the control and Cont + Mel100-treated plants (Figure 8A). Under drought stress, ROS, lipid peroxides, PPO, Fo, and osmotic solutes; different enzymatic antioxidants like SOD, POD, CAT, APX, and PAL; non-enzymatic antioxidants like phenolics and flavonoids; and the total antioxidant activities, increased significantly (Figure 8A), whereas in both the control and the Cont + Mel100-treated plants, these phytochemical trails were at the lowest level. In addition, applied melatonin increased all growth parameters and phytochemical trials, but decreased the ROS, MDA, PPO, and Fo values (Figure 8A), as compared to the drought-stressed plants.

Through heatmap hierarchical clustering, it was observed that three distinguished clusterings were formed among the six treatments, and showed that the drought treatment was separated from the control and the melatonin-treated plants, except for the Cont + Mel50 treatment. In addition, heatmap clustering analysis among all morphological and physiological trials also showed that 100 µM melatonin performed better than other melatonin treatment, as compared to the drought treatment. PCA is a multivariate statistical analysis that could simplify and reduce the dimensionality of high-dimensional complex data, while preserving the original information to the greatest extent. PCA also showed similar results in the heatmap hierarchical clustering analysis. PCA was performed with morphological and physiological trials of all treatments, to evaluate the melatonin effects under drought stress. Two principal components PC1 and PC2 were extracted by PCA to be 70.12% and 27.82%, respectively (Figure 8B). Based on both PC1 and PC2, the six different treatments were clearly distinguished and showed that drought treatment was significantly different from the control and the melatonin treatments had different concentrations. Highly negative correlation for H_2_O_2_, O_2_^•−^, MDA, PPO, and Fo was established in both the PC1 and PC2, whereas all other morphological and physiological trials showed positive correlation only in the PC2 component analysis. In addition, it was also observed that the PCA performed three distinguished clusterings among the morphological and physiological trials, which was the same as the findings of the heatmap analysis (Figure 8A,B). However, PC1 showed that the control and the Cont + Mel100 treatments were clearly separated from other treatments, but were relatively close to D + Mel100. On the other hand, PC2 showed that D + Mel100 was clearly separated from drought and other melatonin treatment, but was relatively close to the control and Cont + Mel100 treatments. The PCA multiple scoring and heatmap hierarchical clustering analysis confirmed that D + Mel100 alleviated the drought effect, which was the nearest to the control (Figure 8A–C).

## 3. Discussion

Drought stress is one of the key abiotic stresses that affects plant growth and biomass production, limiting their physiological, biochemical, and molecular mechanisms (i.e., photosynthesis, protein metabolism, lipid synthesized, etc.) [1,2,27,28]. Our study showed that all plant growth parameters were significantly deteriorated in drought-treated plants with respect to the control (Table 1). These consequences might be owing to the declination of photosynthesis, increased evapotranspiration, decreased cell turgidity, limited CO_2_ assimilation due to stomatal closure, and finally, inhibited cell division under drought stress [29,30]. The biological mechanism of melatonin in plants is not yet fully elucidated, and no clear receptor has been recognized so far. There is strong evidence that melatonin is a growth promoter that can increase tolerance against oxidative stresses [16]. In this experiment, the extent of drought-induced growth inhibition in Tartary buckwheat plants was significantly ameliorated in drought-stressed plants treated with different concentrations of melatonin (Table 1). Endogenous melatonin produced in plants act as a growth regulator or potent antioxidant [16,17]. Additionally, exogenous melatonin has been shown to stimulate the plant antioxidant defense system, due to its powerful direct antioxidant effect [15]. One of the principal strategies identified for melatonin function in plants is the interaction among melatonin, serotonin, and another plant growth regulator, especially auxin, because of their structural homology. There is evidence that melatonin can be linked to the receptor of auxin and acts directly as auxin agonists, increasing cell division and expansion, and ultimately increases plant biomass [31]. One of the vital role of melatonin is the regulation of antioxidant defense in plants, which helps plants in conferring various abiotic stresses including drought [19]. The results of the current study are corroborated to previous research findings of Campos et al. [18] and Liang et al. [3], who reported that the exogenous melatonin amplified the drought tolerance of plants and melatonin acted as a possible release agent for the improvement of plant height, root length, stem circumference, SFW, RFW, SDW, RDW, and leaf number under abiotic stresses [15,31,32,33,34].

Photosynthesis is a principal physiochemical process in which vital photosynthetic pigment like Chl plays a key role in the photosynthetic light reaction, by absorbing energy-rich photon to synthesize plant organic compounds for maintaining plant life activities [32]. In our study, we noticed that the Chl content effectively declined under drought stress (Figure 2A,B). The reduction in Chl content under drought stress possibly occurs due to the impaired synthesis of a major pigment complex of Chl encoded by the genes belonging to the CAB family or an oxidative injury on the lipid and protein structure of chloroplast [35]. Our results were supported by the findings of Campos et al. [18] and Sharma et al. [36], suggesting that melatonin applied on a stressed plant enhanced the photosynthetic pigment, and delayed Chl degradation and leaf chlorosis. Studies regarding the role of melatonin on chloroplast functions often established its positive effect under different abiotic stresses [3,15,18,32,36]. In our research, exogenous melatonin increased Chl content compared to the drought-treated plants (Figure 2A,B), indicating the defensive role of melatonin on Chl content against drought stress. A possible reason might be that melatonin induced activation of *CAB* gene, which is associated with Chl biosynthesis, as well as reduction of the *PAO* gene, which is a key gene in Chl degradation [37,38]. The superiority of melatonin in photosynthetic pigments might be combined with antioxidant utility, as they are known to react with ROS and induce antioxidant activities like SOD, POD, CAT, APX, phenolic, and flavonoid contents (Figure 5 and Figure 6) [19,37].

In addition, analysis of Chl fluorescence was also carried out, which is widely used as a powerful, fast, and dependable technique for monitoring the photosynthesis of plants under stress conditions [39]. This method can expose how much photosystem II (PII) is used for energy absorption by plant Chl under stress. As shown in Figure 2, the Fo value (fluorescence level) increased probably due to the fully oxidized Qa (plastoquinone electron acceptor pool), and the Fv/Fm (maximum quantum efficiency) value reduced when exposed to the drought stress [3,28,36]. The inhibition in photosynthetic activity under drought conditions occurs due to an imbalance between the captured light and their utilization in plant tissues. However, exogenous melatonin treated in the drought stress plants declined the Fo values, whereas, the Fv/Fm ratio increased over the drought-stressed plants (Figure 2C,D). Previous experimental results corresponded with our data, confirming that exogenous melatonin improved the efficiency of photosynthetic apparatus, under stress conditions [3,32,36].

Leaf stomata are known as the key pore to control CO_2_ and water vapor transport, although these functions might be affected by some abiotic factors like atmospheric CO_2_ concentration, water status, temperature, and light [40]. One of the major techniques for plants to improve the water economy is to reduce transpiration through stomata closure. This process was concomitant with a decrease of photosynthesis rate by prevention of CO_2_ entrance into mesophyll cell [41]. Water stress directly declines the availability of CO_2_ in the cells of plants, by restricting diffusion through stomata and decreasing the CO_2_ level in mesophyll by bringing changes in the leaf carbon metabolism and photochemistry [42]. Therefore, declination in photosynthesis under drought stress might be partly ascribed to stomatal closure. In this study, we investigated stomatal properties by using SEM (Figure 1B,C) and observed that the stomatal length, width, and aperture decreased, but stomatal density increased under drought stress with respect to the control (Figure 1B,C and Figure 3). These results might be ascribed to the accumulation of abscisic acid under drought stress, which promotes stomata closure to stop water loss through the evapotranspiration from the leaf under drought stress [43]. In contrast, foliar application of melatonin on drought-stressed plants increased the stomatal density, length, width, and aperture, as compared to drought treatment (Figure 1B,C and Figure 3). Plants have self-developed strategies to prevent oxidative damage by reducing the osmotic potential of the cell by increasing the osmotic solute contents like soluble sugar, soluble protein, and Pro [28,44], and maintaining RWC in the leaves of plants under drought stress (Table 1 and Figure 4A–C). Therefore, these results suggested that exogenous melatonin might have the ability to maintain cell turgor and stomatal opening, through a higher accumulation of osmotic solutes (Figure 4A–C) and maintained RWC (Table 1) in plant leaves, under drought stress.

In the current study, drought stress markedly declined the RWC (Table 1), which might be due to a blockage of water transport from the roots to the shoots, through mesophyll cell turgidity and low leaf water potential, thicker leaf tissue or reduction of soil moisture [30,44]. The RWC in melatonin-treated drought stress treatments was kept closer to the control (Table 1), which was corroborated by a previous study conducted by Su et al. [34]. It was documented that the application of melatonin could prevent water loss through/from leaves by increasing the thickness of cuticle and spongy tissues [28]. According to Cui et al. [44], exogenous melatonin potentially deals with drought-stressed plants by maintaining the water balance and the turgor of plant cells.

Plants also follow a potential strategy to adapt stress conditions by maintaining osmotic adjustment through the production of low molecular weight substances known as osmolytes, such as Pro, soluble sugar, soluble protein, etc. Proline is a familiar osmoprotectant, which has the capacity to protect the cell membrane, protein, and the DNA structure, from ROS-induced damage by quenching of a single oxygen and scavenge OH^•^ [45]. We observed that the Pro content in drought and melatonin-treated drought treatments was significantly increased as compared to the control plants (Figure 4A), where the increase in the Pro content was much higher in melatonin plus drought treatments than in drought alone. Many research findings also established that the accumulation of Pro increased under water stress [3,18,36], and melatonin promoted the production of Pro to cope up with the damaging effect of drought [18,28,36], that are in support of our observed results.

Soluble sugar also plays a significant role in the metabolic process and osmotic balance of cells [46], and protects macromolecules and cell membrane by acting as an ROS scavenger [47,48]. Results showed that the content of soluble sugar was significantly enhanced in a plant grown in both melatonin and drought treatment, as compared to the control (Figure 4B), which might be due to the prevention of denaturation of protein by interacting with membranes and proteins through hydrogen bonds [49].

The content of soluble protein under water stresses was significantly reduced in comparison to the control (Figure 4C). In contrast, the soluble protein content was remarkably augmented in plants under melatonin-treated drought conditions, when compared to drought alone (Figure 4C). Meng et al. [28] and Liang et al. [3] also showed similar results, suggesting that drought stress declined soluble protein content by inducing protein hydrolysis and restraining protein synthesis. In contrary, exogenous melatonin application could inhibit protein degradation and promote new protein synthesis. However, we assumed that exogenous melatonin stimulated the production of osmotic solute contents, which increased the ability of osmotic adjustment and the water retention capacity of plant cells, regulating stomatal movement by scavenging ROS under drought stress.

A general effect of water stress is the generation of excessive ROS in plant cells through deregulation, overflow, or even change of the electron transport chain in the mitochondria and the chloroplast [50], the exciting pigment in thylakoid membranes, through the photosynthesis and respiration process under stress condition [2]. In our study, the concentration of H_2_O_2_, O_2_^•–^, and MDA increased under drought stress (Figure 4D–F). By contrast, foliar application of melatonin in drought-stressed plants significantly decreased H_2_O_2_, O_2_^•–^, and MDA concentration (Figure 4D–F) by acting directly as an antioxidant or by stimulating enzymatic and non-enzymatic antioxidant activities (Figure 5 and Figure 6). It is well-known that melatonin can effectively scavenge environmental-stress-induced ROS through strengthening of the plant defense system by increasing antioxidant enzyme activities [15,18]. Additionally, melatonin itself was also approved as a potential antioxidant with a high capacity to scavenge ROS [17]. In this context, our study data were in accordance with the findings of previous experiments [15,28,32,36].

To sustain under adverse environmental conditions, plants maintain an equilibrium between the formation and detoxification of ROS through antioxidative defense, which consists of enzymatic and non-enzymatic antioxidants [8,14,15]. In our experiment, all studied enzymatic antioxidants, i.e., SOD, POD, CAT, and APX, increased under drought stress, as compared to the control (Figure 5). Melatonin supplementation increased SOD, POD, CAT, and APX activities over drought-treated plants (Figure 5). Melatonin mediated increase in SOD, POD, CAT, and APX protected plants from ROS-induced oxidative injury and, thus, enabled them to survive under drought stress. Many studies reported the effectiveness of melatonin to facilitate plant survival and growth by improving the ROS scavenging defense mechanisms under a water-deficit condition; and the transcription levels of genes coding SOD, POD, CAT, and APX, were upregulated by the exogenous melatonin and significantly increased the expression level of these enzymatic antioxidant activities [15,28,36,51,52].

PAL acts as the major enzyme for the conversion of L-phenyalanine into trans-cinnamic acid in the phenylpropanoid pathway, which subsequently produces phenolic compounds like phenols and flavonoids [13,53,54]. Our results revealed that PAL activity improved under stress conditions relative to the control plants (Figure 6A) [12]. In the melatonin-treated drought stress treatments, increased PAL activity was observed as compared to the untreated melatonin drought-stressed plants, which eventually facilitated the accumulation of total phenolic as well as flavonoid contents (Figure 6A). Sharma et al. [25] also observed an enhanced PAL activity in tomato fruits after melatonin treatment, which was in line with our results.

PPO is another phenolic enzyme that is involved in plant defense against stress conditions by oxidizing phenols to quinines [55]. PPO expression is reported to be induced under various abiotic stress [12], reflecting plant tolerance against stress. In the present study, drought stress increased the PPO expression level, as compared to the control plants, whereas, treatments with applied exogenous melatonin in drought-stressed plants declined PPO activity in relation to plants treated with drought alone (Figure 6B). These results were similar to previous results of Gao et al. [56], who observed the potential of melatonin in ameliorating the oxidative injury to plants under abiotic stress.

Phenolic compounds like phenol and flavonoid are the secondary metabolites [24], which are predictably produced under abiotic stresses [12], and both phenol and flavonoid have a significant role in the assuagement of oxidative damage by detoxifying ROS [12,27]. In our experiment, we quantified a higher total phenol and flavonoid in drought-stressed plants than in the control plants (Figure 6C,D). It further effectively increased the phenol and flavonoid content, in the respective treatments, over the drought-alone treatment (Figure 6C,D). Our findings were in line with the results of Caser et al. [27], who also reported a higher total phenol and flavonoid content in the drought-treated plants than in the control. Additionally, applied exogenous melatonin acted as a signal molecule in drought-stressed plants by significantly enhancing these secondary metabolites to accelerate plant resistance against oxidative damage. These consequences might be due to the increased PAL activities (Figure 6A) in the phenylpropanoid pathway [13,57]. The content of the phenolic compounds exhibited a significant positive correlation (*p* ≤ 0.01) with increasing PAL activities (Figure 7). Previous research findings also proved that drought stress [12] or melatonin application [57] increased the accumulation of phenolic compounds by increasing the PAL activity in the plant leaves.

In this study, it was shown that FRAP and the free DPPH radical scavenging activity increased in the drought alone and melatonin plus drought treatments, as compared to the unstressed plants (Figure 6E,F). Our results were in harmony with the results of Cui et al. [44] and Ye et al. [58], suggesting that exogenous melatonin application boosted stress tolerance by improving the plant’s total antioxidant activities. Phenol and flavonoid contents exert an important role in the plant’s total antioxidant activities. We also observed a positive correlation among the phenolic compounds, PAL, FRAP, and DPPH (Figure 7), corroborating the findings of Dykes [59], who observed the existence of PAL-induced higher phenolic compounds in the plant, leading to an improvement of the plant defense system.

Pearson’s correlation analysis, PCA, and clustering were carried out to explore the interactive relations between the indices and their principal components to acclimatize the plants under drought stress. In our study, correlation analysis showed that ROS and MDA positively correlated with enzymatic and non-enzymatic antioxidants, DPPH, FRAP, Pro, soluble sugar, the Fo values, and stomatal density, whereas, it negatively correlated with all growth parameters, stomata properties (except stomatal density), soluble protein, and RWC (Figure 7). A heatmap is a visual technique that can be used to elucidate complex relations between multiple indices collected from various treatments. It is often useful to combine a heatmap with hierarchical clustering, which is a way of arranging items in a hierarchy, based on the distance or similarity between them. This combined approach (Figure 8A) could clearly identify the significant differences between the treatments among all morphological and physiological traits in this study. PCA biplot analysis (Figure 8B), however, could explore the relative contributions of the indices to the clustered groups. For the purpose of evaluating the effect of melatonin supplementation on plants to enhance the tolerance to drought stress, hierarchical clustering, PCA, and PCA multiple scoring analysis, clearly revealed that 100 µM melatonin could effectively mitigate oxidative damaging effects by enhancing the enzymatic, non-enzymatic antioxidants, and regulating the RWC and the osmotic adjustment.

## 4. Materials and Methods 

The experiment was conducted in the College of Agronomy, Northwest A & F University, Yangling, China (34.29°N and 108.35°E), where temperature, relative humidity, light intensity, and light cycle were 25/18 °C (day and night), 60%, 150 mol m^−2^ s^−1^ photosynthetically active radiation, and 14/10 h (day/night), respectively. The surface of the Tartary buckwheat seeds was disinfected with 0.1% mercuric chloride for 10 min, washed with distilled water, and soaked overnight with deionized water. After this, the seeds were placed in a petri-dish with two layers of wetted filter paper for germination at 28/20 °C temperature and a 16/8 h day/night. Germinated seeds were then transferred into a plastic pot (15 × 15 cm), which was filled with garden soil and vermiculated at a ratio of 1:2.

At the 5th true leaves stage, drought stress was provided by maintaining a 20% field capacity (FC), whereas well-watered (80% FC) pots were used as the control. The required moisture content of all pots was maintained by following the weighing method. To observe the impact of melatonin, the treatments were divided into the following six groups—(i) control (80% FC) (Cont), (ii) control and 100 µM melatonin (Cont + Mel100) (iii) drought (20% FC) (D), (iv) drought stress, and 50 µM melatonin (D + Mel50), (v) drought and 100 µM melatonin (D + Mel100), and (vi) drought and 200 µM melatonin (D + Mel200). All pots were well watered at 80% FC up to 5th leaves initiation, after the drought stress was initiated. The melatonin was sprayed at dark condition, two days before starting drought stress and was continued up to 17 days, with two days interval. Three replications were kept for each treatment, and 10 plants represented each biological replication. In order to investigate the antioxidant defense system, the 2nd to 4th leaves from the tip of the plant were collected, instantly frozen in liquid nitrogen and stored in −80 °C.

### 4.1. Plant Growth Parameter

To detect the changes in plant growth, a meter scale was used for the height and the root length. Vernier calipers were used to measure the diameter of the stem, and a digital weight machine was used to measure the fresh and dry weight of those plants. The plant samples were oven-dried at 80 °C for 72 h to a constant weight, and the dry weight was noted.

### 4.2. Chlorophyll Content and Photosynthesis Activity 

The method followed by Lichtenthaler and Wellburn [60] was used to determine the Chl content. The fresh leaf sample (0.1 g) was chopped into small pieces, soaked in 80% acetone (10 mL) and kept in the dark for 24 h. After this, the filtrates were run in a spectrophotometer to measure the absorbance at 645 and 663 nm, and finally the pigment concentrations were calculated by using the following formula:

Chl a (mg g^−1^ FW) = [12.7(OD_663_) − 2.69(OD_645_)] × V/(1000 × W) (1)

Chl b (mg g^−1^ FW) = [22.9(OD_645_) − 4.68(OD_663_)] × V/(1000 × W) (2)

The Chl fluorescence of leaves was recorded by using a portable modulated MINI PAM-II/B fluorescence meter (HeinZWalZ GmbH Fffeltrich, Germany). These measurements were conducted on dark-adapted plant leaves, using a saturated PPFD (peak at 650 nm) of 3000 µM m^−2^ s^−1^, which was provided by six LED arrays. Fo (initial fluorescence level) and Fv/Fm (photosystem II efficiency) values of the photosystem were measured in the second fully expanded top leaves of the Tartary buckwheat plant [61].

### 4.3. Leaf Ultrastructure

In order to observe the stomata, the second leaves were taken from the tip of the twigs and cut into small pieces (about 1 mm) from the middle rib of leaves to confirm the sample uniformity. Immediately after harvesting, the leaf samples were fixed with 0.2 M phosphate buffer (pH 6.8) with glutaraldehyde solution (4%), and was stored at 4 °C until further use. The segmented leaf samples were then washed four times using 0.1 M phosphate buffer (pH 6.8), was dried out with a series like 30%, 50%, 70%, 80%, 90%, and 100% ethanol solution, and was treated 2 times with isoamyl acetate. After this, a critical point dryer (Emitech K850, Quorum, UK) was used for drying the treated samples and sprayed metal was used for analyzing the leaf stomata with S4800 FESEM (Hitachi Ltd., Tokyo Japan) [28].

### 4.4. Relative Leaf Water Content and Osmotic Solutes

The method of Su et al. [34] was followed to measure relative water content (RWC). Fresh weight (FW) of the leaves (third and fourth) were recorded and were then immediately soaked in deionized water in a petridish, to saturate in a dark condition, for 24 h. Leaf surface water was gently removed with a paper towel, and the turgid weight (TW) was noted. Leaf samples were oven-dried for 72 h at 80 °C, to the constant weight, for determination of the dry weight (DW). The formula used to estimate RWC is as follows:

RWC (%) = [(FW − DW) × 100]/[TW − DW](3)

Leaf soluble protein content was quantified following the method of Bradford [62]. For determination of soluble protein, 0.1 g coosassie brilliant blue G–250 was added to 95% ethanol (50 mL) and 100 mL of 85% phosphoric acid, and the volume was made upto 1 L. A total of 0.2 mL of the enzyme extract was mixed with 0.8 mL distilled water and 5 mL coosassie brilliant blue G–250 reagent, and was vortexed for 30 sec. The absorbance of the prepared solution was recorded at 595 nm in a spectrophotometer, against a reagent blank. The total soluble protein content was calculated using a bovine serum albumin standard curve.

Soluble sugar content was estimated following the anthrone method, as described by Buysse and Merckx [63]. A total of 0.1 g of fresh leaves were cut into small pieces and soaked in 15 mL of deionized water, over-night in dark condition, then boiled for 30 min, until a brown color developed. After cooling, the extract was filtered and deionized water was added to make the volume upto 25 mL. Then, 0.5 mL of the extract sample was mixed with 1.5 mL deionized water, 5 mL of 98% sulfuric acid and 0.5 mL anthrone reagent. Mixture solution was boiled again in a water bath for 1 min and cooled in ice bath. The absorbance was measured at 630 nm against blank, and glucose was used to prepare the standard curve for soluble sugar measurement.

Leaf Pro content was assayed, following the method described by Bates et al. [64]. Fresh leaf samples (0.2 g) were homogenized using 3% sulfosalicylic acid (5 mL) and centrifuged at 12,000 rpm for 15 min at 4 °C. Then, 2 mL plant extract was mixed with 2 mL ninhydrin and 2 mL acetic acid. After this, composite supernatants were boiled for 30 min and 5 mL toluene was added after cooling and kept overnight in the dark condition. The absorbance was recorded through the spectrophotometric method at 520 nm, against toluene, as blank. Standard curve was made with known L-proline for calculation of proline concentration and was expressed as mg g^−1^ FW.

### 4.5. Determination of ROS Activity and MDA Content

To determine H_2_O_2_ concentration, 0.1 g fresh leaf samples were homogenized using 0.1% trichloroacetic acid (TCA) on ice bath and centrifuged at 10,000× *g* for 20 min at 4 °C, 1 mL of 1 M potassium iodide, and 0.5 mL of 10 mM phosphate buffer (pH 7.0) solution were mixed with 0.5 mL supernatant, followed by incubation, and was subjected to measurement of absorbance spectrophotometrically at 390 nm. A standard curve was made with known H_2_O_2_ concentration for calculation of H_2_O_2_ concentration, and was expressed as μ mol g^−^^1^ FW [32].

For quantification of O_2_^•−^, MDA, 0.3 g fresh samples were homogenized with 10 mL of 50 mM phosphate buffer (pH 7.8) on ice bath and centrifuged 2 times at 10,000 x g for 15 min at 4 °C. The resultant leaf extract was preserved at 4 °C for further enzymatic analyses. O_2_^•−^ was estimated according to the method outlined by Elstner and Heupel [65]. A total of 0.5 mL extract solution was mixed with 0.5 mL of 50 mM sodium phosphate buffer (pH 7.8) and 10 mM hydroxylamine hydrochloride (0.1 mL), and was incubated at 25 °C for 1 hr. Then, the incubated solution was mixed with 1 mL 17 mM sulfanilamide and 7 mM naphthylamine, and was again incubated at 25 °C for 20 min. After incubation, the absorbance reading was taken in a spectrophotometer at 530 nm. O_2_^•−^ concentration was calculated with known NaNO_2_ standard curve and expressed as μ mol g^−1^ FW.

Thiobarbituric acid assay was used to estimate the MDA content using the procedure of Han et al. [66], with slight modifications. A total of 2 mL extract solution was added to 2 mL of 0.5% thiobarbituric acid (dissolved with 15% TCA) and boiled at 95 °C for 30 min, then instantly cooled in an ice bath and centrifuged at 4000× *g* for 10 min. The absorbance was measured at 450, 532, and 600 nm, respectively.

### 4.6. Enzymatic Antioxidant Activities

SOD activity was evaluated by calculating its capability to inhibit nitrotetrazolium (NBT) photochemical reduction to the blue formazan by superoxide radicals at 560 nm, following the method used by Giannopolitis and Ries [67]. The reaction solution (6.4 mL) was prepared using 0.75 mM NBT, 50 mM sodium phosphate buffer (pH 7.8), 100 µM EDTA, 130 mM L-methionine, and 20 µM riboflavin, and was mixed with 0.2 mL enzyme extract (taken from MDA and O_2_^•−^ extractant) and 1 mL distilled water (1.2 mL for the blank sample). The tubes were kept in light (only blanks were kept in dark condition) for 20 min, until color development, then covered with a black cloth to stop the reaction.

POD activity was assayed using the guaiacol oxidation method [68], For determination of POD activity, the reaction solution was prepared with 50 mL of 50 mM sodium phosphate buffer (pH 6.0), 5 mM methoxyphenol (28 µL), and 19 µL of 30% H_2_O_2_. A total of 0.02 mL supernatant was mixed with 3 mL reaction solution and the final solution was run into a spectrophotometer to read absorbance at 470 nm.

CAT was measured according to the method used by Chance and Maehly [69], with some modifications. Reaction solution was prepared using 0.1 mM H_2_O_2_ and 100 mM sodium phosphate buffer (pH 7.0) at 4:1. Then, 0.1 mL supernatant was added to 2.5 mL reaction solution and was run in a spectrophotometer three times, after every 1 min interval, and CAT activity was measured by changes in absorbance at 240 nm for 3 min.

Evaluation of the APX activity was done on the basis of H_2_O_2_-dependent oxidation of ascorbate, and the decrease in absorbance was measured at 290 nm [70]. The reaction solution was made with 500 mL of 50 mM sodium phosphate buffer (pH 7.0), 0.04718 g ascorbate (0.54 mM), and 11.2 µL of 30% H_2_O_2_ (0.2 µM). A total of 0.1 mL supernatant was mixed with 2.9 mL reaction solution and absorbance was measured spectrophotometrically at 290 nm.

### 4.7. Contents of Secondary Metabolites Enzymatic and Non-Enzymatic Antioxidant Activity and Total Antioxidant Capacity

PAL activity was estimated according to the previously described method by Zucker [71]. A total of 0.4 mL supernatant (from MDA extractant) was mixed with 0.02 M phenylalanine (2 mL) and 4 mL of 0.1 M sodium borate buffer (pH 8.8), and was kept at 30 °C for 1 h, in a dark condition. After incubation, 0.4 mL of 6 N HCl was added to stop the reaction and filtered. The absorbance reading of the prepared filtrate was recorded using a spectrophotometer at 290 nm.

The activity of PPO was evaluated using the method followed by Nicoli et al. [72], 1 mL of crude extract (extract with 50 mM sodium phosphate buffer, pH 7.8) was mixed with 3 mL of 20 mM caffeic acid, and 1.5 mL of 100 mM buffer (Na_2_HPO_4_/KH_2_PO_4_), pH 7.0, and was incubated for 10 min at 30 °C. After incubation, 0.5 mL of 20% CCl_3_.COOH was added to stop peroxidation of the mixed substrate, and the final filtrate was used for reading the absorbance in a spectrophotometer, at 370 nm.

For determination of the activity of flavonoid, total phenol, DPPH radical, and ferric reducing antioxidant power (FRAP), plant samples were extracted with 6 mL of 80% ethanol and centrifuged at 12,000× *g* for 15 min at 4 °C and was preserved at 4 °C. The Folin-Ciocalteu colorimetric method was used with slight modification for determining the phenolic content [73]. A total of 1 mL extracted sample was added to 0.75 mL Folin-Ciocalteu regent, 0.25 mL sodium carbonate (7.5%), and 1 mL distilled water. The mixed solution was incubated for 90 min at 30 °C, using a water bath. Absorbance reading was taken at 765 nm after the development of the blue color.

Flavonoid content was calculated by the AlCl_3_ colorimetric method [74]. A total of 0.25 mL supernatant was diluted with 0.25 mL ethanoic extractant and vortexed with 5% NaNO_2_ (0.3 mL) and was allowed to stand for 6 min. After this, the solution was mixed with 0.3 mL AlCl_3_ (10%) and kept for 5 min, then 2 mL of 1 M NaOH was mixed and the absorbance was noted after 15 min at 510 nm.

The activity of DPPH free radical scavenging of leaf sample was estimated using the method outlined by Chen and Ho [75]. The supernatant (0.2 mL of 80% ethanol extractant) was mixed with 3.8 mL ethanol solution (80%) of DPPH (0.1 mM) radical, and was incubated for 30 min at room temperature, after vortexing for 1 min. The absorbance of mixed sample (A_sample_) was taken at 517 nm against ethanol blank, and a negative control (A_control_) was measured after mixing the DPPH solution with 0.2 mL ethanol solvent. Ascorbic acid was used as a reference drug. DPPH discoloration percentage was calculated by the following formula: DPPH discoloration percentage = (1− A_sample_ / A_control_) × 100.

The antioxidant ability was determined by measuring FRAP [32]. The FRAP method counts on the reduction of TPTZ (2,4,6-tri-pyridyl-s-triazine)-Fe3+ complex to the TPTZ-Fe2+ complex. The FRAP reagent was prepared by mixing 300 mM acetate buffer (sodium acetate with glacial acetic and distilled water) at pH 3.6 with 20 mM of FeCl3 (dissolved in water) and 10 mM of TPTZ (dissolved in 40 mM of HCl), in the ratio of 10:1:1. Three milliliters of a working FRAP reagent were warmed to 37 °C. Then, 200 µL of sample extract and 300 µL of deionized water were added to the FRAP reagent and the absorbance was taken at 593 nm in a spectrophotometer. FRAP values were expressed as mmol Trolox (6-hydrixy-2,5,7,8-tetramethylchroman-2 carboxylic acid) equivalents (TroloxE) in 1 g FW of leaves.

### 4.8. Statistical Analysis

SPSS software version 20 was used for ANOVA and LSD analysis. Most data were expressed as mean ± standard error. Correlation analysis was done by the statistic software SPSS 2020 and the Heatmap by Origin 2019 software. A hierarchical cluster analysis was performed by the R software. Duncan’s multiple range test (*p* ≤ 0.05) was used to measure the significant differences among the samples.

## 5. Conclusions

**Figure 9:**
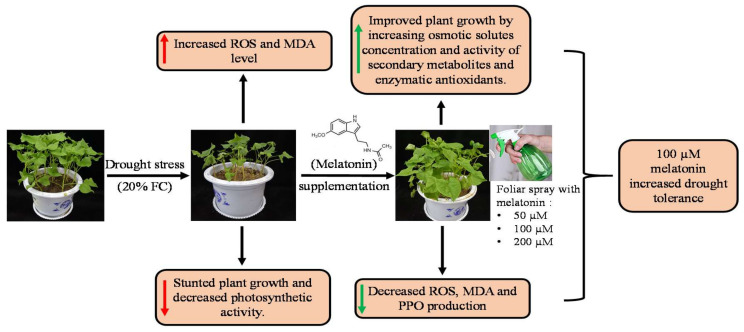
Summary of the mechanism of melatonin-induced drought stress tolerance in Tartary buckwheat.

In the current study, the application of 100 µM melatonin could enhance the capacity of ROS scavenging maximally of Tartary buckwheat plants, by stimulating enzymatic, non-enzymatic, and secondary metabolite enzymatic antioxidant activities, as well as by increasing osmoregulatory solutes that improved the water status in leaves, leading to increased plant growth and development under drought stress. Moreover, melatonin could stimulate the photosynthetic system to avoid ROS-induced oxidative damages by regulating stomatal movement through the osmotic adjustment, and retained the balanced water, improving light energy absorption by the increasing Chl pigments and electron transport in PSII (Figure 9). Since melatonin is a safe and low-priced indoleamine, this might be used as a consistent, practical, and cost-effective plant bio-stimulant for alleviating the effect of drought stress. Our study results suggest that melatonin can efficiently be used to improve plant tolerance to drought, as well as contribute to a potential growth stimulator in crop production.

## Figures and Tables

**Table 1:** Effect of melatonin supplementation on the growth parameters and the relative water content of Tartary buckwheat plants under drought stress.

Treatment	Shoot Height (cm plant^−1^)	Root Length (cm plant^−1^)	No. of Leaves (No. plant^−1^)	Shoot Fresh Weight (g plant^−1^)	Root Fresh Weight (g plant^−1^)	Shoot Dry Weight (g plant^−1^)	Root Dry Weight (g plant^−1^)	Stem Girth (mm plant^−1^)	Relative Leaf Water Content (%)
Cont	23.61 ± 1.27 ^a^	24.33 ± 0.70 ^a^	14.33 ± 0.84 ^a^	3.80 ± 0.40 ^a^	0.228 ± 0.01 ^a^	0.460 ± 0.02 ^a^	0.046 ± 0.002 ^a^	3.77 ± 0.27 ^a^	85.74 ± 1.03 ^a^
Cont + Mel100	23.72 ± 1.62 ^a^	25.00 ± 0.25 ^a^	14.67 ± 0.51 ^a^	3.99 ± 0.36 ^a^	0.236 ± 0.01 ^a^	0.468 ± 0.04 ^a^	0.047 ± 0.002 ^a^	3.87 ± 0.39 ^a^	85.36 ± 1.73 ^a^
D	11.61 ± 0.34 ^d^	12.94 ± 0.39 ^d^	6.34 ± 0.33 ^d^	1.33 ± 0.52 ^d^	0.072 ± 0.00 ^d^	0.216 ± 0.01 ^d^	0.022 ± 0.001 ^d^	2.05 ± 0.04 ^d^	66.15 ± 0.84 ^e^
D + Mel50	13.17 ± 1.33 ^cd^	15.61 ± 1.43 ^cd^	7.33 ± 0.58 ^cd^	1.57 ± 0.02 ^cd^	0.081 ± 0.01 ^cd^	0.246 ± 0.01 ^cd^	0.024 ± 0.002 ^cd^	2.37 ± 0.05 ^cd^	70.90 ± 0.66 ^d^
D + Mel100	19.84 ± 1.01 ^b^	20.83 ± 1.61 ^b^	10.11 ± 0.11 ^b^	2.91 ± 0.14 ^b^	0.167 ± 0.01 ^b^	0.367 ± 0.02 ^b^	0.038 ± 0.002 ^b^	3.13 ± 0.09 ^b^	80.92 ± 1.26 ^b^
D + Mel200	15.45 ± 0.43 ^c^	17.32 ± 1.03 ^c^	8.33 ± 0.19 ^c^	2.10 ± 0.12 ^c^	0.102 ± 0.01 ^c^	0.293 ± 0.01 ^c^	0.028 ± 0.002 ^c^	2.83 ± 0.01 ^c^	76.24 ± 1.21 ^c^

Here, Cont: Control, 80% field capacity (FC); D: Drought, 20% FC; Mel50: 50 µM melatonin; Mel100: 100 µM melatonin and Mel200: 200 µM melatonin. Values of all data are represented as mean ± standard error, with three replications. Means values indicated with different letters refer statistically significant differences at *p* ≤ 0.05, according to Duncan’s multiple range test.

## Supplementary Materials

The following supplementary material is available online, Figure S1: The effect of foliar applied melatonin on DPPH radical Scavenging activity (DPPH EC_50_ values) in Tartary buckwheat leaves under drought stress. Here, Cont: Control, 80% field capacity (FC); D: Drought, 20% FC; Mel50: 50 µM melatonin; Mel100: 100 µM melatonin and Mel200: 200 µM melatonin. Values of all data are representing as mean ± standard error with three replications. Means values indicated with different letter refer statistically significant differences at *p* ≤ 0.05 according to Duncan’s multiple range test.
