# Supplementary material for: Exogenous Melatonin Modulates the Physiological and Biochemical Mechanisms of Drought Tolerance in Tartary Buckwheat (Fagopyrum tataricum (L.) Gaertn)
Source: Molecules. 2020 Jun 18;25(12):2828. doi: 10.3390/molecules25122828 (PMC7355475; doi:10.3390/molecules25122828)
Supplement: Supplementary file 1 [file molecules-25-02828-s001.pdf]

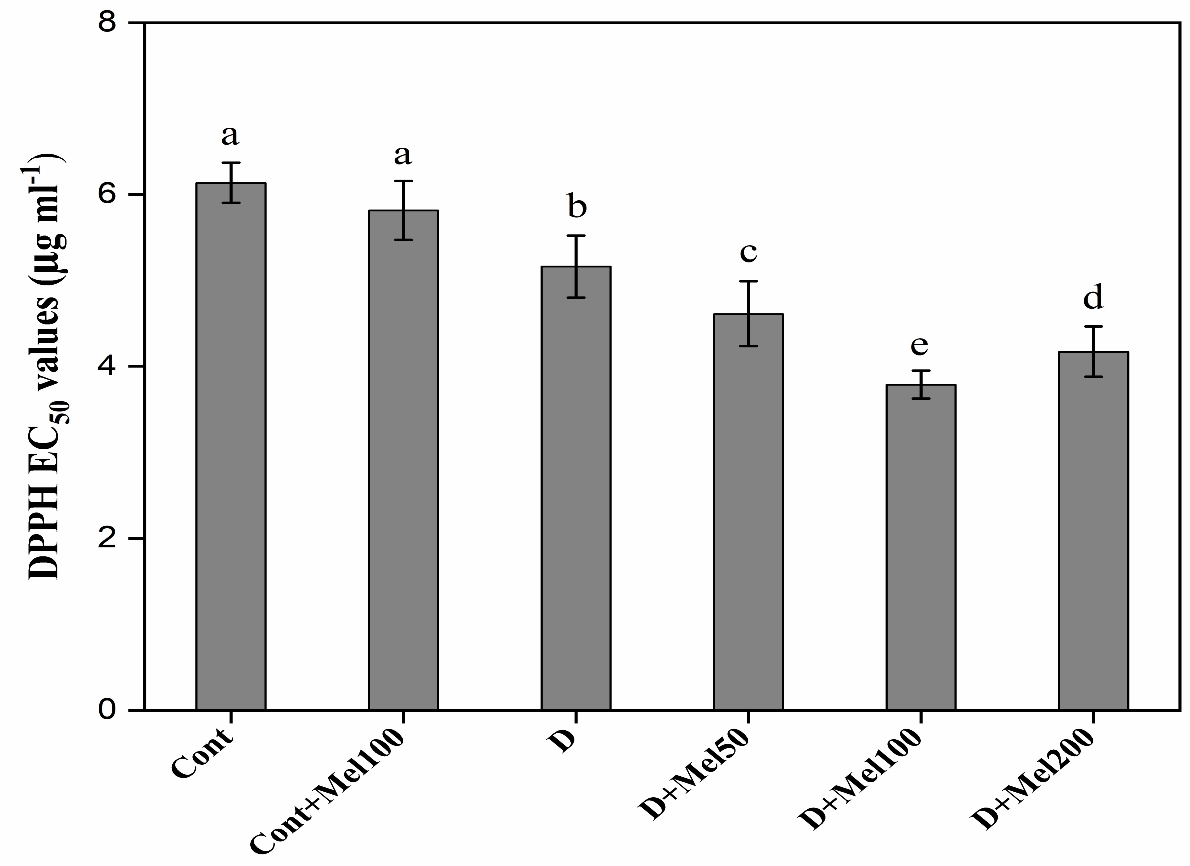

Figure S1. The effect of foliar applied melatonin on DPPH radical Scavenging activity (DPPH EC_{50} values) in Tartary buckwheat leaves under drought stress. Here, Cont: Control, 80% field capacity (FC); D: Drought, 20% FC; Mel50: 50 μM melatonin; Mel100: 100 μM melatonin and Mel200: 200 μM melatonin. Values of all data are representing as mean ± standard error with three replications. Means values indicated with different letter refer statistically significant differences at $p \leq 0.05$ according to Duncan's multiple range test.
